# Protocol for the surgical and large bore procedures in malignant pleural mesothelioma and radiotherapy trial (SMART Trial): an RCT evaluating whether prophylactic radiotherapy reduces the incidence of procedure tract metastases

**DOI:** 10.1136/bmjopen-2014-006673

**Published:** 2015-01-09

**Authors:** Amelia O Clive, Paula Wilson, Hazel Taylor, Anna J Morley, Emma de Winton, Niki Panakis, Najib Rahman, Justin Pepperell, Timothy Howell, Timothy J P Batchelor, Nikki Jordan, Y C Gary Lee, Lee Dobson, Nick A Maskell

**Affiliations:** 1 Respiratory Research Unit, North Bristol NHS Trust, Southmead Hospital, Bristol, UK; 2 Academic Respiratory Unit, School of Clinical Sciences, University of Bristol, Bristol, UK; 3 University Hospitals Bristol NHS Trust, Bristol, UK; 4 Research Design Service South West, Bristol, UK; 5 Royal United Hospital, Bath, UK; 6 Oxford University Hospitals NHS Trust, Oxford, UK; 7 Musgrove Park Hospital, Taunton, UK; 8 Plymouth Hospitals NHS Trust, Plymouth, UK; 9 University Hospitals Bristol NHS Foundation Trust, Bristol, UK; 10 Centre for Asthma, Allergy and Respiratory Research, School of Medicine and Pharmacology, University of Western Australia, Perth, Australia; 11 South Devon Healthcare NHS Foundation Trust, Torbay, UK

## Abstract

**Introduction:**

Patients with malignant pleural mesothelioma (MPM) may develop painful ‘procedure tract metastasis’ (PTM) at the site of previous pleural interventions. Prophylactic radiotherapy has been used to minimise this complication; however, three small randomised trials have shown conflicting results regarding its effectiveness. The surgical and large bore procedures in malignant pleural mesothelioma and radiotherapy trial (SMART Trial) is a suitably powered, multicentre, randomised controlled trial, designed to evaluate the efficacy of prophylactic radiotherapy within 42 days of pleural instrumentation in preventing the development of PTM in MPM.

**Methods and analysis:**

203 patients with a histocytologically proven diagnosis of MPM, who have undergone a large bore pleural intervention (thoracic surgery, large bore chest drain, indwelling pleural catheter or local anaesthetic thoracoscopy) in the previous 35 days, will be recruited from UK hospitals. Patients will be randomised (1:1) to receive immediate radiotherapy (21 Gy in 3 fractions over 3 working days within 42 days of the pleural intervention) or deferred radiotherapy (21 Gy in 3 fractions over 3 working days given if a PTM develops). Patients will be followed up for 12 months. The primary outcome measure is the rate of PTM until death or 12 months (whichever is sooner), as defined by the presence of a clinically palpable nodule of at least 1 cm diameter felt within 7 cm of the margins of the procedure site as confirmed by two assessors. Secondary outcome measures include chest pain, quality of life, analgaesic requirements, healthcare utilisation and safety (including radiotherapy toxicity).

**Ethics and dissemination:**

The trial has received ethical approval from the Southampton B Research Ethics Committee (11/SC/0408). There is a Trial Steering Committee, including independent members and a patient and public representative. The trial results will be published in a peer-reviewed journal and presented at international conferences.

**Trial registration number:**

ISRCTN72767336.

**Strengths and limitations of this study**

- Suitably powered multicentre, randomised controlled trial of prophylactic radiotherapy in malignant pleural mesothelioma.
- Robust 1 year patient follow-up.
- All large bore pleural interventions are eligible, including indwelling pleural catheters.
- Small bore chest tubes excluded.

## Introduction

Malignant pleural mesothelioma (MPM) is an aggressive tumour, which is universally fatal. In 2012, there were 2535 mesothelioma deaths in the UK alone and the incidence is predicted to increase.^1^ ^2^ In order to obtain a robust histological diagnosis and to manage symptomatic pleural effusions, patients commonly undergo a number of diagnostic and therapeutic pleural interventions during the disease course.^3^ ^4^

As a complication of iatrogenic instrumentation of the pleural cavity, tumour can spread to the site of previous interventions, resulting in procedure tract metastases (PTMs), which can be intensely painful.

Mesothelioma is sensitive to radiation therapy in vitro,^5^ but its use as a radical treatment is limited by unacceptable toxicity. However, prophylactic radiotherapy to pleural intervention sites can be given with minimal side effects and a small randomised controlled trial (RCT) was performed in 1995 by Boutin and colleagues to evaluate its efficacy. None of the 20 patients randomised to receive prophylactic radiotherapy (21 Gy in 3 fractions over 3 days within 15 days of the procedure) developed a PTM, but 8 of the 20 patients (40%) in the untreated arm did.^6^

Based on this small study, prophylactic radiotherapy to procedure tracts in mesothelioma became accepted practice worldwide.^4^ ^7–9^ However, the very high rate of PTM in the control arm of the Boutin study was felt to be more than that seen in routine clinical practice, particularly for patients undergoing small bore pleural procedures such as image-guided biopsies or simple pleural fluid aspirations.^10–12^ Additionally, as prophylactic radiotherapy can be burdensome for patients so soon after their diagnosis (requiring up to 4 hospital attendances) and may be associated with some side effects, it was felt further evidence should be obtained to validate its routine clinical use.

Two further RCTs were therefore undertaken; the results of which called into question what had become routine clinical practice.^13^ ^14^ Bydder *et al*^13^ randomised 43 patients with MPM (who had undergone 58 recent small and large bore pleural procedures) to receive a smaller dose of prophylactic radiotherapy (10 Gy in 1 fraction) or no radiotherapy. No significant difference in the occurrence of PTM was identified (7% in the radiotherapy arm vs 10% in the control arm).

These findings were mimicked in another small UK-based RCT evaluating 21 Gy in three fractions of radiotherapy given over 3 days within 21 days of the pleural intervention. Of 61 patients recruited, 7 patients (23%) developed a PTM in the radiotherapy arm, compared with 3 patients (10%) in the control arm.

These conflicting trial results have led to clinical equipoise regarding the benefits of prophylactic radiotherapy and calls from the mesothelioma community for a suitably powered RCT to conclusively establish its role.^15^ In a recent UK survey, 75% of responding centres offered prophylactic radiotherapy, but treatment protocols and patient selection varied greatly between institutions.^15^ Additionally, the advent of chemotherapy for mesothelioma^16^ and the increasing use of indwelling pleural catheters (IPCs) for management of malignant pleural effusion in mesothelioma has resulted in uncertainty regarding the use of prophylactic radiotherapy in these contexts.^17^ ^18^ This study was designed to specifically address these questions with a suitably powered, randomised, controlled trial.

## Methods and analysis

The surgical and large bore procedures in malignant pleural mesothelioma and radiotherapy trial (SMART Trial) is a multicentre, prospective, RCT. The trial is sponsored by North Bristol NHS Trust and coordinated by The Respiratory Research Unit at North Bristol NHS Trust. Data management is undertaken by the Oxford Respiratory Trials Unit. The trial is registered on the International Standardised Randomised Controlled Trial Registry (ISRCTN72767336) and funded by the National Institute for Health Research (NIHR), Research for Patient Benefit (RfPB) Programme. The study is included in the NIHR Clinical Research Network portfolio (ID: 11023). The trial will be conducted in accordance with the Declaration of Helsinki and Good Clinical Practice (GCP).

The primary research question is to evaluate whether prophylactic radiotherapy prevents PTM following large bore pleural procedure in MPM.

The secondary research questions are:

In MPM:

1. Does prophylactic radiotherapy lead to differences in patient symptoms and quality of life indices as compared to radiotherapy given in the event a PTM develops?
2. What proportion of PTM are symptomatic (ie, painful) and to what extent is this modulated by giving prophylactic radiotherapy?
3. In a subgroup of patients with IPCs, is prophylactic radiotherapy effective in reducing PTM?
4. Does prophylactic radiotherapy cause toxicity and impact on the quality of life of patients?
5. Is deferred radiotherapy (given when a nodule develops) as effective as prophylactic radiotherapy at controlling symptoms?
6. What is the patient experience of immediate and deferred radiotherapy?
7. What are the health economic implications of giving prophylactic radiotherapy as compared to deferred radiotherapy in this patient group?

### Setting

**Figure 1:**
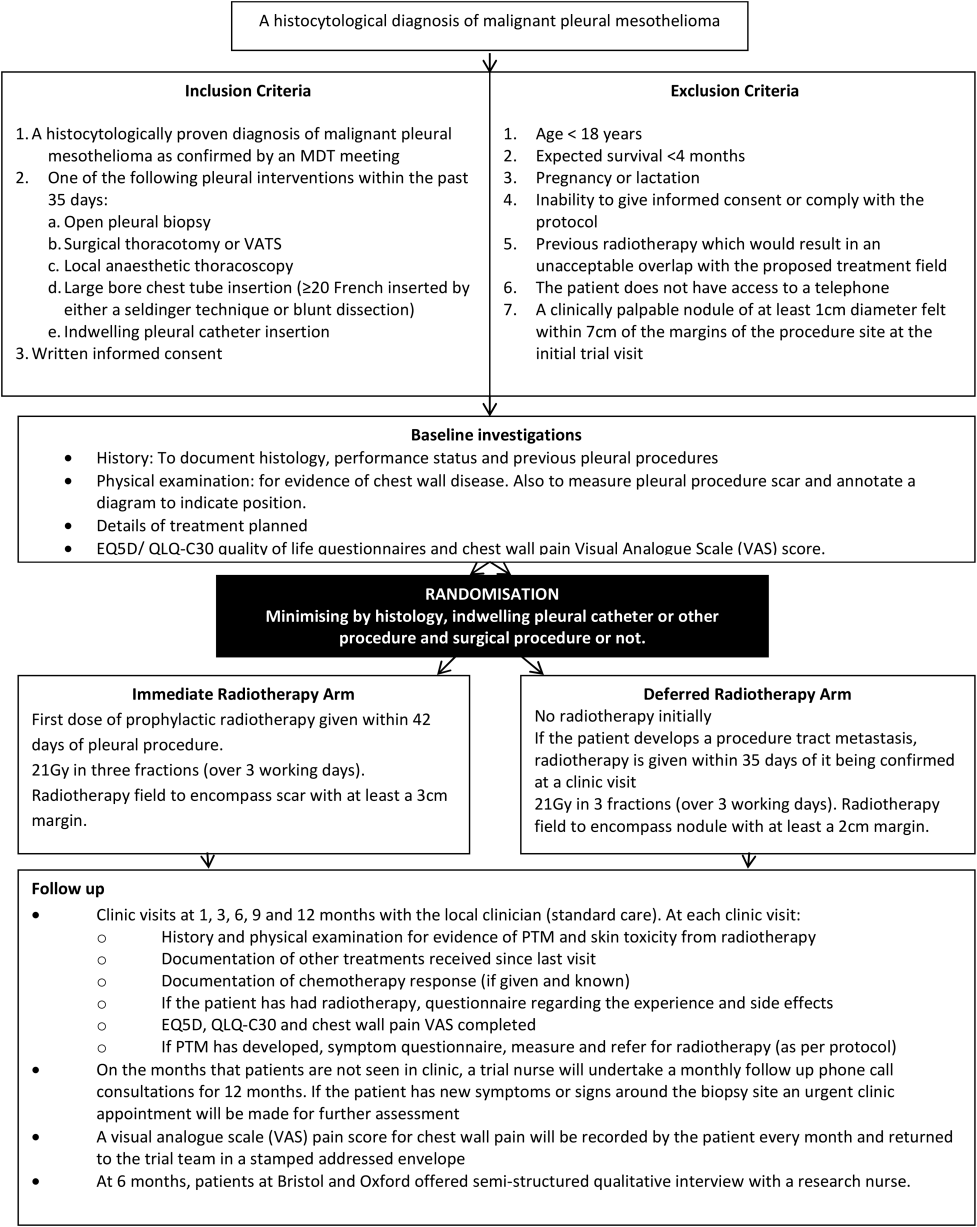
Trial overview flow diagram (MDT, multidisciplinary team; VATS, video-assisted thoracoscopic surgery).

Two hundred and three patients with a histocytologically proven diagnosis of MPM, who have undergone a large bore pleural intervention in the preceding 35 days, will be recruited from UK hospitals (see online supplementary appendix 1 for details of recruiting centres). Patients will be randomised to receive either immediate radiotherapy (within 35 days of their pleural intervention) or deferred radiotherapy (in the event that the patient develops a PTM) and followed up until death or a year (whichever is sooner). The study flow diagram is shown in figure 1.

### Participant screening and selection

All patients discussed at the regional mesothelioma multidisciplinary team (MDT) meetings will be identified as potential trial candidates. Consecutive, eligible patients will be invited to participate and will be provided with a patient information sheet (PIS; see online supplementary appendix 2). Patients can only be enrolled into the SMART Trial once.

### Inclusion criteria

1. A histocytologically proven diagnosis of mesothelioma, as confirmed by a MDT meeting.
2. One of the following pleural interventions in the past 35 days:
  - Open pleural biopsy;
  - Surgical thoracotomy or video-assisted thoracoscopic surgery (VATS);
  - Local anaesthetic thoracoscopy;
  - Large bore chest tube insertion (greater or equal to 20 Fr inserted by either a seldinger technique or blunt dissection);
  - IPC insertion.
3. Written informed consent.

### Exclusion criteria

1. Age <18 years;
2. Expected survival <4 months;
3. Pregnancy or lactation;
4. Inability to give informed consent or comply with the protocol;
5. Previous radiotherapy which would result in an unacceptable overlap with the proposed treatment field;
6. The patient does not have access to a telephone;
7. A clinically palpable nodule of at least 1 cm diameter felt within 7 cm of the margins of the procedure site at the initial trial visit.

### Informed consent

A doctor will confirm patient eligibility prior to consent being taken. Patients will be given at least 24 h to consider the PIS and time to ask questions prior to written informed consent being taken by a trial doctor, nurse or radiographer. The consent form can be viewed in online supplementary appendix 3.

### Randomisation

Following informed consent, patients will be randomly assigned (1:1) to receive either immediate prophylactic radiotherapy (within 42 days of the pleural intervention) or deferred radiotherapy (given if the patient develops a PTM).

Treatment allocation will be performed over the telephone by UKCRC Oxford Respiratory Trials Unit. The randomisation sequence will be generated using a validated, online randomisation service (Sealed Envelope, London, UK; http://www.sealedenvelope.com). Minimisation with a random component will be used to reduce the baseline between-group differences.

The minimisation factors are:

- Histological tissue type of mesothelioma (epithelioid only or other);
- IPC or other pleural procedure;
- Surgical procedure (ie, open pleural biopsy, thoracotomy or VATS) or non-surgical procedure.

Patients and clinicians will not be blinded to treatment allocation; however, the data analysis will be conducted in a blinded fashion.

### Standard care

All patients will be discussed in an MDT meeting. If appropriate, patients will be referred to the local oncologist for discussion and consideration of their treatment options. Aside from prophylactic radiotherapy, other treatments offered to patients will be guided by clinical need and are at the discretion of the patient's clinicians.

Ongoing clinical review will either take place in oncology or respiratory clinic. Patients requiring assistance from other services, for example, the surgeons, palliative care team or hospice, will be referred when needed by the clinical team. Patients who require radiotherapy for another indication (other than prophylactic radiotherapy as part of the trial or for treatment of a PTM) can be treated at the discretion of the local oncologist.

Co-enrolment in other clinical trials will be discussed on an individual basis, but will only be considered if compliance with both protocols can be ensured. Patients can withdraw from the trial at any time without their clinical care being affected.

### Interventions

The full trial-specific procedure for radiotherapy can be found in online supplementary appendix 4.

#### Immediate radiotherapy

For patients in the immediate (prophylactic) radiotherapy arm, radiotherapy should be given within 35 days of the pleural procedure for which the patient has been randomised. Under exceptional circumstances, the first fraction may be postponed for up to 42 days but a reason must be clearly stated.

The treatment to be given will be 21 Gy in three fractions over 3 working days. The preferred procedure is to treat the patient using electrons of appropriate energy to treat the chest wall to at least 90% (using bolus if necessary). Alternatively, kilovoltage (kV) photons (minimum 220 kV) can be used if the depth dose to the chest wall is adequate. Megavoltage photons (MV) can be used if clinically indicated. A single direct beam will be used in the majority of cases.

The volume to be treated must be acceptable to the treating clinical oncologist and the treatment area should be no less than 7 cm in any one direction.

For pleural procedures other than IPCs:

- Suggested clinical target volume (CTV): chest drain and surgical sites/scars with at least a 3 cm margin;
- Suggested planning target volume (PTV): CTV+0.5 cm if using kV photons or CTV+1 cm if using electrons;
- The total radiotherapy field will be equivalent to the PTV.

For patients with an IPC in situ:

- Suggested CTV: pleural puncture site, the whole of the catheter tract and the skin exit site with at least a 3 cm margin;
- Suggested PTV: CTV+0.5 cm if using kV photons or CTV+1 cm if using electrons;
- The total radiotherapy field will be equivalent to the PTV.

If patients in the immediate radiotherapy arm develop a PTM, further radiotherapy treatment to the site is at the discretion of the treating oncologist. Details of the relationship of the PTM to the prophylactic radiotherapy field will be recorded.

#### Deferred radiotherapy

If patients in the deferred radiotherapy arm are diagnosed with a PTM, 21 Gy in three fractions of radiotherapy will be given within 35 days of the PTM being diagnosed. The dose, technique, energy and beam arrangement will be the same as for the immediate radiotherapy arm and the treatment volume must be acceptable to the treating clinical oncologist.

- Suggested CTV: palpable nodule with at least a 2 cm margin;
- Suggested PTV: CTV+0.5 cm if using kV photons or CTV+1 cm if using electrons;
- The total radiotherapy field will be equivalent to the PTV.

### Data collection and management

#### Randomisation visit

Clinical data will be collected at the randomisation visit. This will include a history and chest wall examination and patients will be asked to complete two quality of life questionnaires (EQ5D and QLQ-C30) and visual analogue scale (VAS) scores to quantify their current degree of chest pain.

#### Follow-up visits

Follow-up visits will be undertaken at 1, 3, 6, 9 and 12 months postrandomisation. These will include a focused history (including details of radiotherapy toxicity, mesothelioma treatments received, analgaesia use and healthcare utilisation) and a chest wall examination to identify PTMs and radiation toxicity. The patient will also be invited to complete quality of life questionnaires (QLQ-C30 and EQ5D), a VAS score for chest pain and patient experience questionnaires regarding radiotherapy or the development of a PTM (if applicable).

#### Telephone follow-up

When patients are not seen in clinic, they will receive a monthly phone call from a research nurse to enquire about symptoms at the intervention site. They will also be invited to complete a chest pain VAS score, which will be returned by post. Should they develop problems at the intervention site, a clinic appointment will be arranged as soon as possible (ideally within 10 days).

#### Semistructured interviews

A small, qualitative substudy will explore patients’ experiences of being in the trial, their perceptions of the treatments and risks involved, to inform the trial results. Participants at the Bristol and Oxford sites will be invited to take part in semistructured qualitative interviews by a research nurse approximately 6 months after randomisation (see online supplementary appendix 5 for interview schedule).

From those who agree to be interviewed, a purposive maximum variation sample will be selected of up to 20 patients. Patients from Bristol and Oxford who develop a tract metastasis at any point after their 6-month follow-up visit will be invited to undertake a follow-up interview.

The data will be analysed using a thematic approach and the themes produced will help to describe participants’ views of the trial and how treatment can be optimised for these patients. This will be published separately to the main trial outcomes.

#### Data management

Clinical Record Forms will be completed by the trial team at the recruiting centre and sent to the Oxford Respiratory Trials Unit. Data will then be entered onto the trial database (OpenClinica clinical trials software). Missing data and data queries will be highlighted to the trial teams on a monthly basis. The Clinical Record Forms will only identify patients using their personal trial identification number (no identifiable patient information).

### Primary outcome

The primary outcome will be the difference in the incidence of development of PTM within 7 cm of the site of pleural intervention within 12 months from randomisation between the study arms.

A PTM will be defined as a clinically palpable nodule of at least 1 cm diameter felt within 7 cm of the margins of the procedure site as confirmed by two assessors. One of the assessors must be a doctor who feels that clinically the nodule is a tract metastasis. The assessors will be doctors, nurses or radiographers who have read the chest wall examination standard operating procedure (SOP) (see online supplementary appendix 6) and feel confident to undertake the examination. In the event of disagreement between the assessors, they will examine the patient together to reach a consensus.

The presence or absence of PTM within 12 months of randomisation will be compared using Fisher's exact test. This was chosen in preference to ‘time-to-event’ analysis for the primary outcome analysis, as prophylactic radiotherapy is not likely to change the speed with which the events occur, but rather prevent them from occurring, thereby violating the necessary assumptions of survival models. Time-to-event data will be modelled using regression analysis, but this will form part of the secondary analysis.

### Secondary outcomes

Specific details regarding all the secondary outcomes can be found in the statistical analysis plan in online supplementary appendix 7. The secondary outcomes will include:

- The change in chest pain VAS scores from randomisation to 12 months postrandomisation between the study arms;
- The change in quality of life (as measured by EQ5D and QLQ-C30) from randomisation to 12 months postrandomisation between the study arms;
- The difference in analgaesia requirements between the study arms;
- The difference in the size, symptom severity and time to development of PTM between the study arms;
- The rate and severity of radiotherapy toxicity;
- The number of serious adverse events (SAEs) related to radiotherapy or a PTM;
- The health economic implications of immediate and deferred radiotherapy;
- The identification of emergent themes from the semistructured interviews.

### Sample size calculation

The sample size calculation was based on internal audit data showing a PTM rate of <2% in those treated with prophylactic radiotherapy who underwent ‘large’ bore procedures, in comparison to a rate of between 8% and 40% in the published literature of patients not undergoing prophylactic radiotherapy.

Assuming the rates of PTM to be 2% in the intervention group and 15% in the ‘control’ group, 180 patients are required to demonstrate a difference between treatment arms with 90% power at the 5% significance level. With an estimated lost to follow-up rate of 3%, this number is increased to 185 patients randomised 1:1. Various power modelling scenarios were considered (see table 1), demonstrating adequate power with varied control and intervention event rates if 203 patients are recruited.

**Table 1:** Sample size calculations for different predicted PTM rates

	Control group As needed radiotherapy					
Intervention group Prophylactic radiotherapy	90% power (α 0.05, 3% loss to FU)			80% power (α 0.05, 3% loss to FU)		
Incidence of PTM at 12 months (%)	12	15	18	12	15	18
1	203	150	118	153	112	88
2	269	185	140	201	140	105
3	357	234	169	266	174	125
4	482	294	203	360	220	152
5	670	375	248	500	281	184

All rates include a projected 3% loss to follow-up. The figures in the shaded cells are those which are achievable within the proposed sample size.

FU, follow up; PTM, procedure tract metastasis.

### Statistical analysis plan

The full statistical analysis plan can be viewed in online supplementary appendix 7. The analysis will be based on intention-to-treat principles.

For continuous outcomes, analyses will adjust for the minimisation factors and the outcome measured at baseline (provided there is no substantial missing baseline data). For binary outcomes, if the number of events allows, the analysis will adjust for the minimisation factors. If major baseline imbalances between the treatment arms are identified, these may also be adjusted for in the regression models.

For missing baseline data, if the numbers are small, median imputation will be used. If the numbers with missing data are large, alternative analysis methods may be used, which do not account for baseline values.

CONSORT data will be presented including the number of patients screened for the study and the numbers randomised. Reasons for exclusions after randomisation will be given.

Analysis for all outcomes will be presented as:

1. The number of participants included in the analysis, by treatment group;
2. A summary statistic for the outcome, by treatment group (eg, mean (SD) or median (2.5th–97.5th centile) for continuous outcomes; number (%) for binary outcomes);
3. A treatment effect, with a 95% CI and p value (2 sided; the significance level set at 5%).

The primary outcome, incidence of PTM within 12 months, will be analysed on an intention-to-treat basis. The proportion of patients developing a PTM (as defined above) within 12 months from randomisation or until death or loss to follow-up (whichever is sooner) will be calculated for both trial arms and compared using Fisher’s exact test. Alternatively, if the number of events allows, logistic regression will be performed, adjusting for the minimisation variables and any substantial baseline imbalances.

A secondary, per protocol analysis will be performed for the primary outcome, excluding patients with major protocol violations.

If numbers allow, the following subgroup analyses will be performed for the primary outcomes:

- By the type of pleural intervention randomised (large bore chest drain, local anaesthetic thoracoscopy, IPC or thoracic surgery);
- By tumour subtype (epithelioid only or other);
- Patients who were alive and in trial follow-up for at least 6 months (yes/no);
- Patients who received chemotherapy for mesothelioma within 12 months from trial entry (yes/no).

Full details of the analysis of the secondary outcomes can be found in statistical analysis plan (see online supplementary appendix 7).

### Changes to the protocol after start of the trial

The trial details documented here are consistent with SMART Trial protocol V.7 (date: 21 August 2013). A summary of the trial amendments can be found in online supplementary appendix 8.

In July 2012, the inclusion criteria were extended to include patients up to 35 days after their large bore pleural intervention (from 28 days) and to lengthen the maximum timeframe within which immediate radiotherapy should be performed to 42 days after the pleural intervention (from 35 days).

### End of the trial

The trial will end once 203 patients have been recruited and all patients have died or completed 1 year of trial follow-up (whichever is sooner).

## Ethics and dissemination

All substantial amendments will be submitted to the ethics committee for their approval prior to implementation (see online supplementary appendix 8).

### Monitoring

As advised by the NCRI Radiotherapy Trials Quality Assurance Group (RTTQA), departments involved in delivering radiotherapy for the trial will be required to provide evidence of an independent audit measurement within the past 5 years for the radiotherapy modalities being used in the trial.

There will be no formal data monitoring committee for this study, as the risk profile of prophylactic radiotherapy is already well established. No interim analysis is planned.

### Safety reporting

Data will be collected at each trial visit regarding any SAEs (as defined by GCP). All SAEs causally related to radiotherapy or a PTM will be reported to the sponsor and followed until they resolve or stabilise.

Radiotherapy toxicities will be recorded at each follow-up visit (according to the Radiation Therapy Oncology Group (RTOG) grading system).

IPC complications will also be recorded at each clinic visit.

### Trial monitoring and oversight

The Trial Steering Committee (TSC) will be responsible for overseeing the progress of the trial and will meet at approximately six monthly intervals. The TSC will include an independent chairperson, independent members, statistician, patient and public representative and members of the trial team from all the main disciplines (respiratory medicine, oncology, palliative care and thoracic surgery).

### Dissemination

The trial will be publicised at regional and national conferences. The final results will be presented at scientific meetings and published in a peer-reviewed journal (authorship will be according to the journal's guidelines). In addition, a lay summary of the study results will be circulated to potentially interested parties (eg, local and national mesothelioma charities and the trial participants).

### Trial status

The trial is currently in follow-up. The first patient was recruited in December 2011 and the final patient was enrolled in August 2014.

## Supplementary Material

Reviewer comments
