# Supplementary material for: Protocol for the surgical and large bore procedures in malignant pleural mesothelioma and radiotherapy trial (SMART Trial): an RCT evaluating whether prophylactic radiotherapy reduces the incidence of procedure tract metastases
Source: BMJ Open. 2015 Jan 9;5(1):e006673. doi: 10.1136/bmjopen-2014-006673 (PMC4289725; doi:10.1136/bmjopen-2014-006673)
Supplement: Reviewer comments [file bmjopen-2014-006673.reviewer_comments.pdf]

PEER REVIEW HISTORY

BMJ Open publishes all reviews undertaken for accepted manuscripts. Reviewers are asked to complete a checklist review form (**http://bmjopen.bmj.com/site/about/resources/checklist.pdf**) and are provided with free text boxes to elaborate on their assessment. These free text comments are reproduced below. Some articles will have been accepted based in part or entirely on reviews undertaken for other BMJ Group journals. These will be reproduced where possible.

ARTICLE DETAILS

**TITLE (PROVISIONAL)**	Protocol for Surgical and large bore procedures in malignant pleural mesothelioma and radiotherapy trial (SMART Trial) – An RCT evaluating whether prophylactic radiotherapy reduces the incidence of procedure tract metastases.
**AUTHORS**	Clive, Amelia; Wilson, Paula; Taylor, Hazel; Morley, Anna; de Winton, Emma; Panakis, Niki; Rahman, Najib; Pepperell, Justin; Howell, Timothy; Batchelor, Tim; Jordan, Nikki; Lee, YC Gary; Dobson, Lee; Maskell, Nick

-This article was not externally reviewed at BMJ Open. Protocols that have been independently assessed prior to submission to BMJ Open are usually fast-tracked to publication on the grounds that further substantial changes will not be possible. This independent assessment will usually be external, independent review for both a substantial grant award from a non commercial or government funder and ethics approval.
